# Evaluation of the antiplasmodial efficacy of synthetic 2,5-diphenyloxazole analogs of compounds naturally derived from *Oxytropis lanata*

**DOI:** 10.1016/j.ijpddr.2024.100540

**Published:** 2024-04-18

**Authors:** Nanang R. Ariefta, Koichi Narita, Toshihiro Murata, Yoshifumi Nishikawa

**Affiliations:** a National Research Center for Protozoan Diseases, Obihiro University of Agriculture and Veterinary Medicine, Inada-cho, Obihiro, 080-8555, Japan; b Faculty of Pharmaceutical Sciences, Tohoku Medical and Pharmaceutical University, 4-4-1 Komatsushima, Aoba-ku, Sendai, 981-8558, Japan

**Keywords:** *2,5-Diphenyloxazoles*, *Antiplasmodial*, *Plasmodium falciparum*, *Plasmodium yoelii*

## Abstract

The persistent prevalence and dissemination of drug-resistant malaria parasites continue to challenge the progress of malaria eradication efforts. As a result, there is an urgent need to search for and develop innovative therapies. In this study, we screened synthetic 2,5-diphenyloxazole analogs from *Oxytropis lanata*. Among 48 compounds, 14 potently inhibited the proliferation of *P. falciparum* strains 3D7 (chloroquine-sensitive) and K1 (multidrug-resistant) in vitro, exhibited IC_50_ values from 3.38 to 12.65 μM and 1.27–6.19 μM, respectively, and were toxic to human foreskin fibroblasts at 39.53–336.35 μM. Notably, Compounds **31** (2-(2′,3′-dimethoxyphenyl)-5-(2″-hydroxyphenyl)oxazole) and **32** (2-(2′,3′-dimethoxyphenyl)-5-(2″-benzyloxyphenyl)oxazole) exhibited the highest selectivity indices (SIs) against both *P. falciparum* strains (3D7/K1), with values > 40.20/>126.58 and > 41.27/> 59.06, respectively. In the IC_50_ speed and stage-specific assays, Compounds **31** and **32** showed slow action, along with distinct effects on the ring and trophozoite stages. Microscopy observations further revealed that both compounds impact the development and delay the progression of the trophozoite and schizont stages in *P. falciparum* 3D7, especially at concentrations 100 times their IC_50_ values. In a 72-h in vitro exposure experiment at their respective IC_80_ in *P. falciparum* 3D7, significant alterations in parasitemia levels were observed compared to the untreated group. In Compound **31**-treated cultures, parasites shrank and were unable to reinvade red blood cells (RBCs) during an extended 144-h incubation period, even after compound removal from the culture. In vivo assessments were conducted on *P. yoelii* 17XNL-infected mice treated with Compounds **31** and **32** at 20 mg/kg administered once daily for ten days. The treated groups showed statistically significant lower peaks of parasitemia (Compound **31**-treated: trial 1 12.7%, trial 2 15.8%; Compound **32**-treated: trial 1 12.7%, trial 2 14.0%) compared to the untreated group (trial 1 21.7%, trial 2 28.3%). These results emphasize the potential of further developing 2,5-diphenyloxazoles as promising antimalarial agents.

## Highlights

• Antiplasmodial evaluation of synthetic 2,5-diphenyloxazole analogs of natural products found in *Oxytropis lanata.*
• Fourteen compounds potently inhibited the proliferation of *P. falciparum* strains 3D7 and K1 in vitro.
• Compounds **31** and **32** exhibited the highest selectivity indices in vitro and significantly decreased parasitemia levels in vivo.

## Graphical abstract

**Figure:**
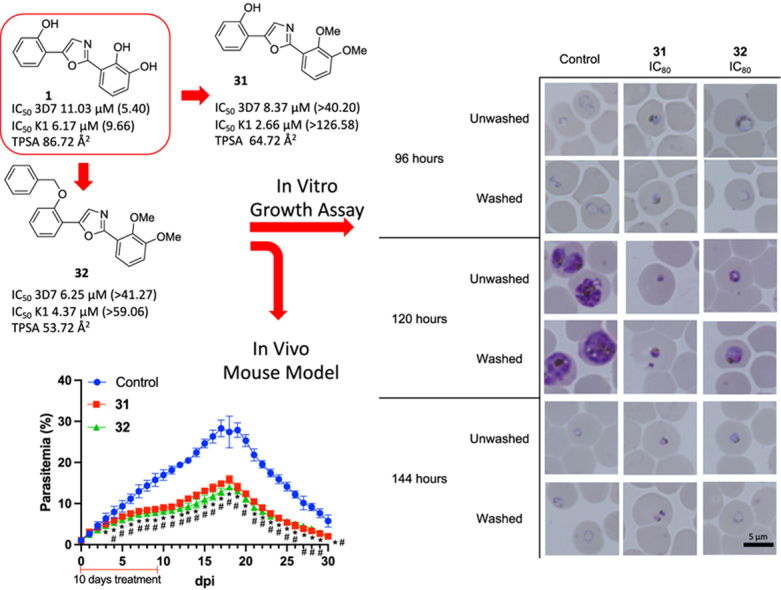

## 1 Introduction

In the last decade, significant advancements have been made in terms of antimalarial drugs and vector control strategies, which has led to a reduction in the impact of malaria. However, this disease remains a persistent affliction for half of the global population (White, 2008). The number of malaria cases, a life-threatening disease caused by *Plasmodium* parasites, increased to 247 million worldwide in 2022 (World Health Organization, 2023). Additional challenges have arisen due to the reduced efficacy of commercially available antimalarial drugs because of the parasite's ability to develop chemoresistance (Mideo et al., 2013). This disturbing phenomenon applies not only to lesser-known medications, as even the well-known artemisinin combination therapy confronts the challenge of chemoresistance (Talisuna et al., 2004). Therefore, the continuous exploration of new antiplasmodial agents and pathways susceptible to applied chemotherapies are needed to develop new antimalarial medicines and therapies.

The main mechanism of many antimalarial drugs involves the blood stages of *Plasmodium* spp. This emphasis has arisen because these stages not only trigger the clinical manifestations of malaria but also facilitate substantial parasite population expansion through repeated cycles of invasion, development, replication, and emergence from red blood cells (Kappe et al., 2010; Skinner-Adams et al., 2012). Dedicated efforts have been directed toward discovering naturally existing compounds possessing antiplasmodial potency. This quest has led to the isolation of diverse active frameworks, including alkaloids, terpenoids, flavonoids, xanthones, quinones, macrocycles, and cyclic peptides (Batista et al., 2009; Kaur et al., 2009; Wells, 2011). Naturally occurring 1,3-oxazole-containing compounds, including 1,3-oxazole fused to peptides, macrolides, polyketides, and benzoxazoles, have been previously investigated and shown to have promising various bioactivities (Chen et al., 2021). The isolation and structural elucidation of 2,5-diphenyloxazoles from the roots of Mongolian *Oxytropis lanata* was previously reported (Banzragchgarav et al., 2016), and some of these compounds exhibited antiprotozoal activities, including trypanocidal activity. Furthermore, in our prior study on the Mongolian compound library, we found that 2-(2′-hydroxy-5′-methoxylphenyl)-5-(2″,5″-dihydroxyphenyl)oxazole and 2-(2′,5′-dihydroxyphenyl)-5-(2″-hydroxyphenyl)oxazole (**4**) from this same plant exhibited antiplasmodial activity (Banzragchgarav et al., 2021). Although several naturally occurring 2,5-diaryloxazoles have been isolated and some have been found to be biologically active, Compounds **1** (2-(2′,3′-dihydroxyphenyl)-5-(2″-hydroxyphenyl)oxazole), **4** (2-(2′,5′-dihydroxyphenyl)-5-(2″-hydroxyphenyl)oxazole), **7** (2-phenyl-5-(2″,5″-hydroxyphenyl)oxazole), and **10** (2-(2′,5′-dihydroxyphenyl)-5-(2″,5″-dihydroxyphenyl)oxazole) were the first naturally occurring 2,5-diphenyloxazoles isolated from *O. lanata*. These compounds possess hydroxy groups on the phenyl ring, which has been reported to be associated with antiprotozoal activity (Narita et al., 2021). Therefore, in the present study, we use the attractive scaffolds of these compound derivatives as the starting point for the development of new antiplasmodial agents.

## 2 Materials and methods

### 2.1 Compounds

**Fig. 1:**
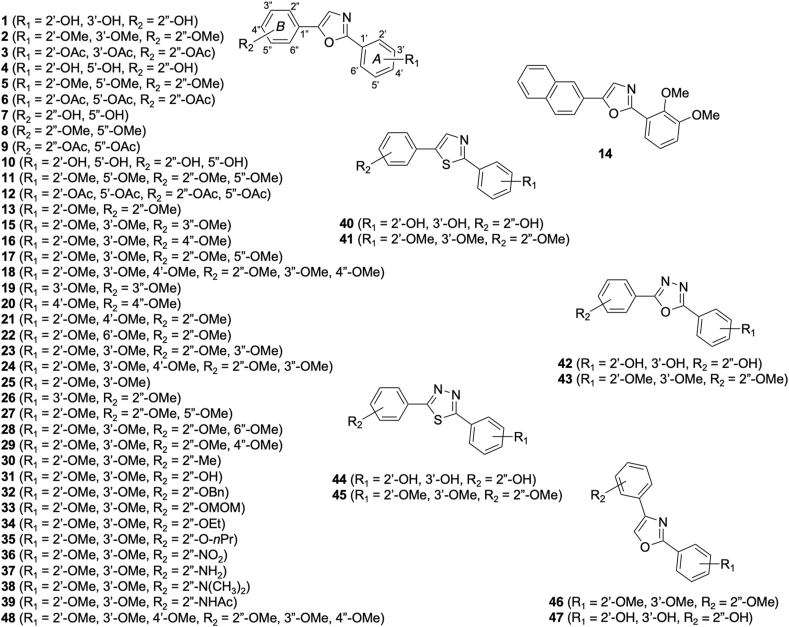
The structures of the 2,5-diphenyloxazoles used in this study.

Forty-eight 2,5-diphenyloxazoles (Fig. 1) were synthesized following a prior study (Narita et al., 2021). Positive controls for antiplasmodial activity included chloroquine diphosphate (CHQ, MW: 515.86 g/mol, CAS: 50-63-5) and artemisinin (ART, MW: 282.33 g/mol, CAS: 63968-64-9), both obtained from Sigma‒Aldrich (St. Louis, MO, USA). As a negative control, DMSO (MW: 78.13 g/mol, CAS: 67-68-5, Wako, Osaka, Japan) was utilized. The highest DMSO concentration used for in vitro testing was 0.01%; at this level, the solvent's presence did not impact parasite proliferation (Naidu et al., 2018).

### 2.2 Parasites

The in vitro study included two human malaria parasites: a chloroquine-sensitive strain (3D7) and a multidrug-resistant strain (K1) of *Plasmodium falciparum*. Additionally, in vivo studies were carried out using a nonlethal malaria rodent model involving *P. yoelii* 17XNL.

### 2.3 In vitro culture of *P. falciparum*

The *P. falciparum* strains 3D7 and K1 were cultivated in complete medium consisting of the following components: Roswell Park Memorial Institute (RPMI)-1640 (Sigma‒Aldrich, St. Louis, MO, USA), 25 mM HEPES (CAS: 7365-45-9, Sigma‒Aldrich, St. Louis, MO, USA), 0.5% (w/v) AlbuMax™ II (lipid-rich BSA, Gibco, Waltham, MA, USA), 24 mM NaHCO_3_ (CAS: 144-55-8, Wako, Osaka, Japan), 184 μM hypoxanthine (CAS: 68-94-0, Wako, Osaka, Japan), and 0.025% (v/v) gentamicin (50 mg/mL, CAS: 1403-66-3, Gibco, Waltham, MA, USA) supplemented with 2% washed human O-(+) erythrocytes, provided by the Japanese Red Cross Society, Hokkaido, Japan. The cultures were maintained in an incubator under specific conditions: 37 °C under 5% CO_2_ and 5% O_2_. The parasitemia levels were monitored using Giemsa-stained thin blood smears (CAS: 51811-82-6, Merck, Darmstadt, Hesse, Germany). Ethical approval for human blood malaria parasite culture was obtained with permit number #2013-04-3 from the ethical committee of Obihiro University of Agriculture and Veterinary Medicine adhering to the ethical standards for research involving human blood samples.

### 2.4 In vitro inhibition of *P. falciparum*

The D-sorbitol method (CAS: 50-70-4, Wako, Osaka, Japan) was employed to synchronize parasites, resulting in the attainment of ≥90% ring-stage parasites. These synchronized parasites were then utilized for the experiments within a 2-h window. The assay was performed as follows. First, 50 μL of synchronized parasites with 0.5% parasitemia and 2% hematocrit were added to individual wells of a 96-well plate. Then, 50 μL of the test compounds was introduced to each well. To determine the IC_50_, each compound or drug was prepared from a 10 mM stock solution (in DMSO) and subjected to twofold dilutions using complete medium. This process resulted in eight different final concentrations ranging from 200 μM to 1.5625 μM or 10 μM–0.039 μM. Wells containing only test compounds and erythrocytes were included for background correction. The 96-well plate was incubated for 72 h (24, 48, and 72 h for ART, CHQ, and Compounds **31** and **32**) at 37 °C under 5% CO_2_ and 5% O_2_. The SYBR Green I-based fluorescence assay was used to detect the growth of *P. falciparum* 3D7 and K1 following a described protocol (Leesombun et al., 2021; Smilkstein et al., 2004). After the designated incubation time, 100 μL of lysis buffer containing 0.02% (v/v) SYBR Green I was added to each well, the contents of the well were mixed by pipetting, and the plate was incubated in the dark at room temperature for 2 h. Fluorescence intensities were measured using a Fluoroskan Ascent instrument (Thermo Fisher Scientific, Waltham, MA, USA) with an excitation wavelength of 485 nm and emission wavelengths at 518 nm. This assay was repeated four times at each concentration, and the resulting inhibitory values represent the average from three independent trials.

### 2.5 In vitro toxicity to human cells

The cytotoxicity of the tested compounds was evaluated using a cell viability assay with human foreskin fibroblasts (HFFs) following established methods (Ariefta et al., 2022; Leesombun et al., 2021). HFF cells are selected for cytotoxicity testing in this study, providing a physiologically relevant model for the evaluation of drug interactions with human cells. The significance of these cells is highlighted by their importance in key biological functions like cell growth, differentiation, and response to oxidative stress (Nadalutti and Wilson, 2020). This designation makes them a suitable model for the investigation of cellular processes and their sensitivity to toxic substances, which is crucial for the early identification of potential adverse effects of drugs during the development process. The reliability of the results is ensured by various reports that have utilized HFF cells in the cytotoxic assessment of antiplasmodial activity (Arnold et al., 2021; Brinner et al., 2005; Choomuenwai et al., 2012).

### 2.6 Prediction of drug-likeness properties

All compounds underwent analysis using two tools: SwissADME (Daina et al., 2017) and pkCSM pharmacokinetics (Pires et al., 2015). These analyses were conducted to predict the drug-likeness properties of the compounds, encompassing both pharmacokinetic and pharmacodynamic profiles.

### 2.7 Structure-activity relationship analysis

Python within a Jupyter Notebook environment was used to examine the correlation between molecular descriptors calculated from SwissADME (molecular weight, number of hydrogen bond acceptors, number of hydrogen bond donors, number of rotatable bonds, topological polar surface area, and partition coefficient) and the biological activity of compounds, represented by base-10 log-transformed IC_50_ values (logIC_50_). The transformation to logIC_50_ was conducted to normalize the distribution of the IC_50_ values, enhancing the applicability and interpretability of linear correlation analyses. The Pearson correlation coefficient (*r*), calculated using the ‘*SciPy. Stats’* library (Virtanen et al., 2020), was used to measure the linear relationship between each descriptor and logIC_50_, with the coefficient ranging from −1 to 1 to indicate perfect negative to perfect positive linear correlations, respectively. The statistical significance of these correlations was assessed through *p*-values, with values < 0.005 indicating significant correlations. Visualization of these findings was facilitated by the ‘*matplotlib’* (Hunter, 2007) and ‘*seaborn’* (Waskom, 2021) libraries, which were used to generate bar plots illustrating the correlation coefficients and their *p*-values. The code of this analysis is provided in the supplementary data.

### 2.8 Effects of in vitro treatments with compounds 31 and 32 on the morphology and parasitemia level of *P. falciparum* 3D7

To ensure uniformity, the parasites underwent a rigorous synchronization process using a two-step sorbitol treatment procedure. This synchronized parasite population was harnessed within a 2-h window for the ring stage, 8–10 h for the trophozoite stage, and 24–26 h for the schizont stage. Subsequent to sorbitol treatment, early trophozoites could be obtained within 6–8 h after the initial sorbitol treatment (within 20 h of development), while early schizonts could be obtained after an additional 16 h of incubation (within 36 h of development). Beyond 36 h, the schizonts mature, causing the rupture of red blood cells (RBCs) and the consequent release of merozoites, which promptly infect new RBCs. Up to the 48-h time point post synchronization, the ring stage becomes observable once more, with trophozoites becoming apparent at 72 h (Hofer et al., 2008; Le Manach et al., 2013).

For the stage-specific examination focusing on the ring and schizont stages (Le Manach et al., 2013), parasites at either the ring or schizont stage were utilized, each with a parasitemia of 0.5% and a hematocrit of 2%. In this analysis, treatment groups were exposed to Compounds **31**, **32**, ART, and CHQ. These compounds were administered at starting doses of 100-fold their respective IC_50_ values, followed by two-fold serial dilutions across 8 concentrations. Wells containing only the test compounds and erythrocytes were included to correct for background activity. The 96-well plates were incubated for 24 h at 37 °C in an atmosphere of 5% CO_2_ and 5% O_2_. The growth of *P. falciparum* 3D7 was detected using a SYBR Green I-based fluorescence assay.

For stage-specific microscopy examination, parasites in the ring, trophozoite, or schizont stage were employed, each with a parasitemia of 0.5% and a hematocrit of 2%, as previously reported (Ariefta et al., 2023). In this assessment, Compounds **31** and **32**, ART, and CHQ were utilized in different treatment groups, and the compounds were administered at doses of 100-, 10-, and 1-fold their respective IC_50_ values. The negative control group consisted of cultures treated solely with complete medium and was designated the no treatment (NT) group. This analysis was repeated three times at each concentration. Following a 24-h incubation period, the culture in a 96-well plate was centrifuged at 1300 *g* for 5 min to settle down the iRBC; then, the supernatants were removed, and the iRBCs were washed with fresh media at the same volume as the initial culture. The centrifugation and washing were repeated 3 times, allowing the compound to be diluted by approximately 1000 times. Subsequently, thin blood smears from each treatment group were prepared using 1 μL of settled iRBCs. The proportion of parasites affected by the drug, including those with both abnormal and retained morphologies, was calculated by examining 100 parasites and counting the number displaying irregular development. Subsequently, the number of abnormal parasites was divided by the total count and then multiplied by 100 to obtain a percentage. The parameters used to define normal and abnormal morphologies can be found in Table S3 and Fig. S1.

In the 72-h compound exposure assay with the compound wash-unwashed procedure, parasites at the ring stage with 0.5% parasitemia and 2% hematocrit were employed. For this evaluation, Compounds **31** and **32** were used at concentrations equivalent to their respective IC_80_ values (Compound **31**: 18.08 μM; hill slope 2.10, Compound **32**: 11.74 μM; hill slope 2.02). These groups were considered the treated groups, while cultures with only complete medium served as untreated groups. The IC_80_ was used to allow observations of parasite morphology under incomplete inhibition, the potential emergence of abnormal morphologies, and the state of parasites following compound removal. This experimental setup was repeated three times for each concentration and time point. Thin blood smears were prepared from each treatment group at 1, 24, 48, or 72 h post incubation. In different culture sets, after 72 h of incubation, the medium was exchanged, and the compounds were subjected to three washes using fresh complete medium. Incubation was then continued either in the presence (unwashed group) or absence (washed group) of the test compounds for up to 144 h. Thin blood smears were consecutively prepared from each treatment group at 96, 120, and 144 h. The smears were visualized using a BZ-900 all-in-one microscope (Keyence BioRevo, Tokyo, Japan). Parasitemia levels were calculated by counting the number and determining the ratio of infected erythrocytes relative to the number of uninfected erythrocytes.

### 2.9 Mice and in vivo infection

Male C57BL/6 mice, aged eight weeks, were obtained from Japan CLEA (Tokyo, Japan) and housed with six per cage in each group. These mice were kept under conditions of 24 °C, 50% relative humidity, and a light-dark cycle of 8 a.m. to 8 p.m. Access to drinking water and food (CLEA Rodent Diet CE-2; Japan CLEA, Tokyo, Japan) was unrestricted. Prior to experimentation, *P. yoelii* 17XNL was recovered from cryopreserved erythrocyte stocks *via* passage in mice. Infection was established through intraperitoneal (i.p.) injection of freshly infected erythrocytes from donor mice (1 × 10^7^ iRBCs/mouse), designated 0 days post-infection [dpi]. Parasitemia levels were evaluated after 2 h by collecting 2 μL of blood from the mouse tail for thin blood smear preparation. Treatment was initiated upon reaching a parasitemia level of 1%. Intraperitoneal administration of vehicle (1 × PBS), Compound **31** (20 mg/kg), or Compound **32** (20 mg/kg) was carried out once daily for ten days (0–9 dpi). Parasitemia in infected mice was observed daily by preparing Giemsa-stained thin blood smears (up to 30 dpi). Hematocrit levels were measured every other day using a Celltac-α MEK-6550 (Nihon Kohden, Tokyo, Japan), with 10 μL of blood collected from the mouse tail for each assessment. The animals were handled and treated in accordance with the Guide for the Care and Use of Laboratory Animals by the Ministry of Education, Culture, Sports, Science, and Technology, Japan. Ethical permission for the animal experiment protocols was secured under permit numbers 21–35 and 23–90 from the ethical committee of Obihiro University of Agriculture and Veterinary Medicine.

### 2.10 Statistical analysis

The IC_50_ or CC_50_ values were determined from three independent experiments by plotting the logarithmic concentration of the compound against the percent parasite inhibition or cell growth inhibition, respectively. Nonlinear regression analysis was conducted using GraphPad Prism 10 (GraphPad Software, Inc., La Jolla, CA, USA). Comparisons between groups were evaluated using either one-way or two-way ANOVA followed by Tukey's or Fisher's multiple comparisons tests. A *p-value* of <0.05 was considered to indicate statistical significance and is denoted by an asterisk or a symbol. The meaning of each symbol is specified in the corresponding figure legend alongside the name of the employed test.

## 3 Results

### 3.1 2,5-Diphenyloxazoles inhibit the multiplication of *P. falciparum* in vitro

The antiplasmodial activity of the 48 synthesized compounds was first evaluated against *P. falciparum* 3D7. Among them, 14 compounds were found to exhibit potent inhibitory activity (Table S1). The antiplasmodial activities of these 14 compounds are summarized in Table 1, and they had IC_50_ values ranging from 3.38 to 12.65 μM and 1.27–6.19 μM for *P. falciparum* 3D7 and K1, respectively. Synthetic compounds that initially occurred naturally, Compounds **1**, **4**, **7**, and **10**, were found to be active against both *P. falciparum* strains. It is important to note that the resistance index values for all 14 compounds identified were below 1. This finding suggests that the scaffold of these compounds is particularly more effective against resistant strain K1. Furthermore, these 14 compounds exhibited CC_50_ values against HFFs ranging from 39.53 to 336.35 μM, allowing the calculation of selectivity indices (SIs) ranging from 2.61 to 41.27 and 3.94–126.58 for *P. falciparum* 3D7 and K1, respectively. Notably, Compounds **31** and **32** exhibited the highest SI values against both *P. falciparum* 3D7/K1, with values of >40.20/>126.58 and > 41.27/> 59.06, respectively. Additionally, these two compounds also exhibited a lower than 3% hemolysis rate at 1000 μM, comparable with the positive controls (see supplementary data Fig. S2). These two compounds were chosen for further study because of their high safety properties based on SI values.

**Table 1:** Summary of the in vitro activities of the 2,5-diphenyloxazole derivatives compared with the positive controls^a^.

Sample	Structure	Molecular Weight (g/mol)	IC_50_*P. falciparum* (μM)			CC_50_ HFFs (μM)	SI	
			3D7	K1	Resistance index		3D7	K1
**1**		269.26	11.03 ± 2.69	6.17 ± 3.33	0.56	59.56 ± 4.84	5.40	9.66
**3**		395.37	9.22 ± 1.68	4.70 ± 2.24	0.51	125.57 ± 31.64	13.61	26.69
**4**		269.26	6.20 ± 4.23	3.74 ± 1.47	0.60	55.36 ± 11.69	8.93	14.81
**7**		253.26	3.85 ± 1.87	1.65 ± 0.72	0.43	54.11 ± 11.00	14.06	32.89
**9**		337.33	3.38 ± 1.46	1.27 ± 0.51	0.38	56.68 ± 18.38	16.79	44.47
**10**		285.26	12.65 ± 7.63	4.94 ± 2.15	0.39	39.53 ± 7.45	3.13	8.00
**12**		453.40	10.23 ± 4.09	4.70 ± 1.69	0.46	67.79 ± 1.39	6.63	14.43
**31**		297.31	8.37 ± 1.28	2.66 ± 1.15	0.32	>336.35	>40.20	>126.58
**32**		387.44	6.25 ± 1.39	4.37 ± 1.70	0.70	>258.10	>41.27	>59.06
**40**		285.32	3.95 ± 1.38	1.74 ± 0.89	0.44	46.30 ± 5.84	11.71	26.60
**42**		270.24	9.53 ± 3.15	6.19 ± 2.66	0.65	52.51 ± 13.19	5.51	8.48
**44**		286.31	6.41 ± 1.16	3.91 ± 1.45	0.61	160.21 ± 62.34	25.01	40.96
**47**		269.26	4.63 ± 1.176	3.13 ± 1.21	0.68	71.02 ± 23.14	15.33	22.68
**48**		401.42	8.01 ± 3.20	4.34 ± 1.89	0.54	96.47 ± 7.31	12.04	22.21
Chloroquine (diphosphate)		515.86	0.026 ± 0.003	0.784 ± 0.073	30.15	20.71 ± 6.80	796.54	26.41
Artemisinin		282.336	0.014 ± 0.003	0.019 ± 0.002	1.36	153.00 ± 30.76	10,928.57	8052.63

a The reported values are presented as the average ± standard deviation (SD) derived from three independent experiments. Abbreviations used: IC_50_: half-maximal inhibitory concentration; CC_50_: half-maximal cytotoxic concentration; Resistance index, the ratio between the IC_50_ values of *P. falciparum* K1 and 3D7; HFFs: human foreskin fibroblasts; SI: selectivity index, a measure of the compound's selectivity for parasite inhibition (IC_50_) relative to its toxicity to human cells (CC_50_).

**Fig. 2:**
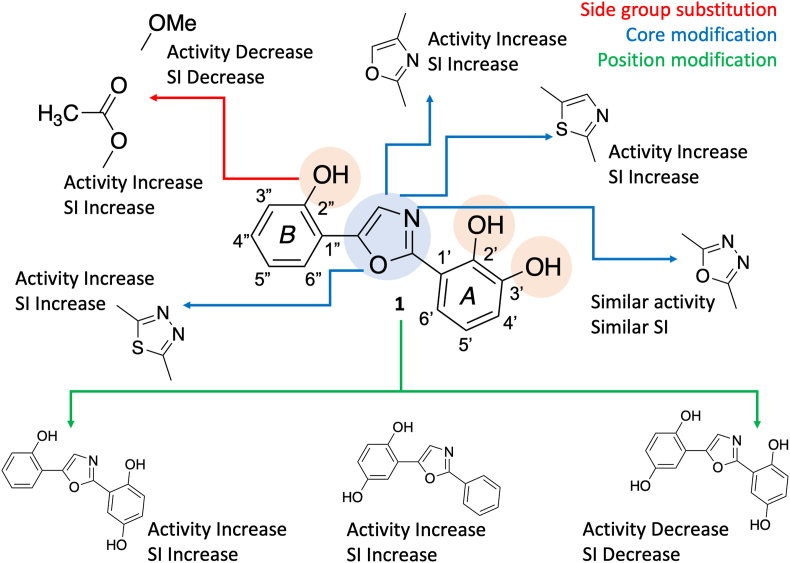
Structure-activity relationship among the 2,5-diphenyloxazoles in this study.

Fig. 2 illustrates the structure-activity relationship (SAR) of a set of closely related compounds, where the significant impact of side group substitution and core modifications on biological activity is underscored. A clear trend was revealed by the SAR analysis, in which the replacement of hydroxyl groups with methoxy or acetyl groups—indicated by red arrows—was found to lead to reduced or increased inhibitory activity, respectively. Consistency in this pattern was observed across compounds that underwent identical group transformations, such as Compounds **1**, **2**, and **3**. The critical role of alterations to the central heterocyclic scaffold, denoted by blue arrows, was suggested to potentially enhance the inhibitory activity. Marked by green arrows, positional modifications were shown to influence activity as well, with a preference for certain structural features or interactions, such as hydrophobic, hydrophilic, or pi-pi stacking interactions, which were notably significant for Compounds **31** and **32**.

**Fig. 3:**
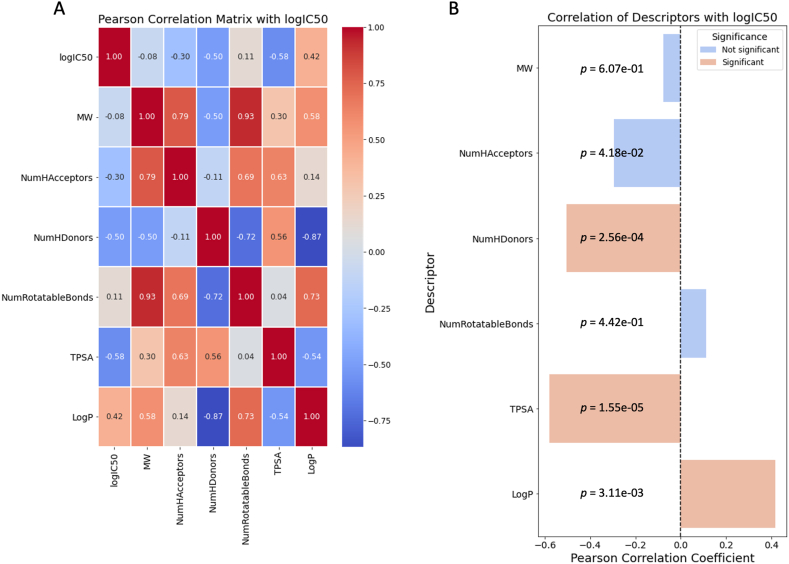
(A) The Pearson correlation matrix of descriptors with LogIC_50_ and (B) correlation of descriptors with LogIC_50_ of tested 2,5-diphenyloxazoles in this study. As indicated in the legend, significance levels are defined by *p*-value <0.005.

A SAR statistical analysis was conducted on all compounds, examining the correlation between molecular descriptors calculated from SwissADME (molecular weight, number of hydrogen bond acceptors, number of hydrogen bond donors, number of rotatable bonds, topological polar surface area, and partition coefficient) and the IC_50_ against *P. falciparum* 3D7. The correlation matrix heatmap (Fig. 3A) provides a visual depiction of the interrelationships between molecular descriptors, with both positive and negative correlations influencing biological activity being delineated. For instance, a strong positive correlation—such as a coefficient of 0.93—was shown by certain descriptors, which might suggest a concerted effect on biological activity. On the other hand, pronounced negative correlations—such as −0.87—were displayed by others, implying an opposing influence on activity. The heatmap was complemented by statistical analysis, which pinpointed specific molecular descriptors that have marked correlations with IC_50_ (Fig. 3B), thereby elucidating their role in the inhibitory potential of the compounds. A robust negative correlation to IC_50_ (*r* = −0.504) was exhibited by the Number of Hydrogen Bond Donors (NumHDonors), accompanied by a highly significant *p*-value (*p* = 2.56e-04), which was indicative of an association between a higher count of hydrogen bond donors and enhanced compound activity. The Topological Polar Surface Area (TPSA), reflecting a molecule's polar characteristics, also demonstrated a significant negative correlation with IC_50_ (*r* = −0.580, *p* = 1.55e-05), indicating that an increased TPSA is favorable for activity. In contrast, LogP presented a positive correlation (*r* = 0.418, *p* = 3.11e-03), indicating that more lipophilic compounds are associated with higher IC_50_ values, suggesting reduced biological activity. This information serves as a guide for refining molecular designs to improve potency.

### 3.2 Prediction of the drug likeness of the 2,5-diphenyloxazoles

All tested compounds, in addition to ART and CHQ, underwent analysis using the SwissADME web server (http://www.swissadme.ch/, accessed on August 23, 2023, all 48 compounds) and the pkCSM-pharmacokinetics web server (http://biosig.unimelb.edu.au/pkcsm/, accessed on August 23, 2023, 14 hit compounds). These analyses were conducted to forecast the drug-likeness, ADME (absorption, distribution, metabolism, excretion), and toxicity of the compounds. The summarized drug-likeness properties of all submitted compounds are provided in Table 2 and Table S2. All compounds were found to have drug-like properties. Because of the close structural similarity among the tested compounds, their ADMET features were found to be similar. Based on these analyses, the following inferences were made. (1) All 14 hit compounds were predicted to be noncarcinogenic according to the AMES toxicity profile. (2) Some of the compounds showed potential hepatotoxicity. (3) Oral rat acute toxicity assessments indicated a high possible concentration at the LD_50_ values, ranging from 2.26 to 3.149 mol/kg. These values vary in terms of the dose tolerance in humans, which range from 0.136 to 0.833 log ml/min/kg. In humans, typically a dose tolerance greater than 0.477 log ml/min/kg is desired. (4) The predicted intestinal absorption of all hit compounds exceeded 84%.

**Table 2:** Predicted pharmacokinetic and pharmacodynamic properties of the 14 hit 2,5-diphenyloxazole derivatives.

Parameter	Compound																
	**1**	**3**	**4**	**7**	**9**	**10**	**12**	**31**	**32**	**40**	**42**	**44**	**47**	**48**	Chloroquine	Artemisinin	Desired value
Molecular Weight (g/mol)	269.26	395.37	269.26	253.26	337.33	285.26	453.4	297.31	387.44	285.32	270.24	286.31	269.26	401.42	319.87	282.336	≤500
No. H-bond acceptors	5	8	5	4	6	6	10	5	5	4	6	5	5	8	2	5	≤10
No. H-bond donors	3	0	3	2	0	4	0	1	0	3	3	3	3	0	1	0	≤5
No. rotatable bond (rotors)	2	8	2	2	6	2	10	4	7	2	2	2	2	8	8	0	≤10
Topological polar surface area (TPSA, Å^2^)	86.72	104.93	86.72	66.49	78.63	106.95	131.27	64.72	53.72	101.82	99.61	114.71	86.72	81.41	28.16	53.99	≤140
Lipophilicity Log *P* octanol/water partition coefficient	2.25	3.14	2.3	2.65	3.3	1.89	3.12	2.98	4.52	2.89	1.9	2.48	2.27	3.34	4.15	2.5	≤5
Water solubility Log S (ESOL) solubility class	−3.64 soluble	−4.04 moderately soluble	−3.64 soluble	−3.80 soluble	−4.04 moderately soluble	−3.49 soluble	−4.05 moderately soluble	−4.04 moderately soluble	−5.56 moderately soluble	−4.13 moderately soluble	−3.28 soluble	−3.76 soluble	−3.64 soluble	−4.43 mederately soluble	−4.55 moderately soluble	−3.42 soluble	Insoluble < −10 < Poorly < −6 < Moderately < −4< Soluble < −2 Very <0< Highly
Metabolism																	
CYP2D6 substrate	No	No	No	No	No	No	No	No	No	No	No	No	No	No	Yes	No	
CYP3A4 substrate	Yes	Yes	Yes	Yes	Yes	No	Yes	Yes	Yes	Yes	Yes	Yes	Yes	Yes	Yes	Yes	
CYP1A2 inhibitor	Yes	Yes	Yes	Yes	Yes	Yes	Yes	Yes	Yes	Yes	Yes	Yes	Yes	Yes	No	No	
CYP2C19 inhibitor	Yes	Yes	Yes	Yes	Yes	Yes	No	Yes	Yes	Yes	Yes	Yes	Yes	no	No	No	
CYP2C9 inhibitor	Yes	Yes	Yes	Yes	Yes	Yes	No	Yes	Yes	Yes	Yes	Yes	Yes	Yes	No	No	
CYP2D6 inhibitor	No	No	No	No	No	No	No	No	No	No	No	No	No	No	Yes	No	
CYP3A4 inhibitor	Yes	No	Yes	No	No	Yes	Yes	Yes	Yes	Yes	No	Yes	Yes	Yes	No	No	
Intestinal absorption (% absorbed)	90.247	92.839	90.158	91.24	97.454	84.212	100	94.32	97.685	89.598	80.473	89.912	90.636	97.545	89.95	97.543	≥30%
Total clearance (log ml/min/kg)	0.348	0.749	0.437	0.617	0.851	0.593	1.207	0.57	0.672	0.303	0.007	0.264	0.406	0.838	1.092	0.98	
BBB permeant	No	No	No	Yes	No	No	No	Yes	Yes	No	No	No	No	No	Yes	Yes	
LD_50_ oral rat acute toxicity (mol/kg)	2.358	2.628	2.492	2.409	2.703	2.475	3.149	2.333	2.515	2.328	2.26	2.297	2.375	2.515	2.85	2.459	
Hepatoxicity	No	Yes	No	No	Yes	No	Yes	No	Yes	No	No	No	No	Yes	Yes	No	
AMES toxicity (act as a carcinogen)	No	No	No	No	No	No	No	No	No	No	No	No	No	No	Yes	Yes	
Max tolerated dose in human (log ml/min/kg)	0.271	0.555	0.268	0.293	0.671	0.752	0.741	0.136	0.78	0.295	0.315	0.323	0.214	0.833	−0.167	0.065	≥0.477 high tolerance
Drug likeness																	
Lipinski (Pfizer)	Yes	Yes	Yes	Yes	Yes	Yes	Yes	Yes	Yes	Yes	Yes	Yes	Yes	Yes	Yes	Yes	
Ghose	Yes	Yes	Yes	Yes	Yes	Yes	Yes	Yes	Yes	Yes	Yes	Yes	Yes	Yes	Yes	Yes	
Veber (GSK)	Yes	Yes	Yes	Yes	Yes	Yes	Yes	Yes	Yes	Yes	Yes	Yes	Yes	Yes	Yes	Yes	
Egan (Pharmacia)	Yes	Yes	Yes	Yes	Yes	Yes	Yes	Yes	Yes	Yes	Yes	Yes	Yes	Yes	Yes	Yes	
Abbott Bioavailability Score	0.55	0.55	0.55	0.55	0.55	0.55	0.55	0.55	0.55	0.55	0.55	0.55	0.55	0.55	0.55	0.55	
Synthesis accessibility score	2.92	3.48	2.92	2.9	3.27	2.99	3.74	3.12	3.67	2.84	2.71	2.82	2.79	3.75	2.75	6.13	from 1 (very easy) to 10 (very difficult)

In conjunction with these predictions, the majority of the compounds were not identified as substrates for CYP2D6, implying a favorable metabolic profile. However, the diverse substrate activity concerning CYP3A4 underscores the need for additional exploration to ascertain their metabolic robustness. Widespread inhibition of key CYP enzymes by the compounds points to a significant drug-drug interaction risk, suggesting a cautious approach in multi-drug regimens. Moderate lipophilicity, indicated by LogP values primarily between 2 and 3, suggests optimal cell membrane permeability, while the solubility classifications from soluble to moderately soluble, as per LogS values, hint at a generally favorable bioavailability profile. Overall, it can be concluded that all of the hit compounds exhibited drug-likeness properties based on the Lipinski (Lipinski et al., 2001), Ghose (Ghose et al., 1999), Veber (Veber et al., 2002), and Egan (Egan et al., 2000) parameters, making them promising candidates for further drug development and evaluation.

### 3.3 Effects of compounds 31 and 32 on the IC_50_ speed, stage specificity, morphology, and parasitemia level of *P. falciparum* 3D7 in vitro

**Fig. 4:**
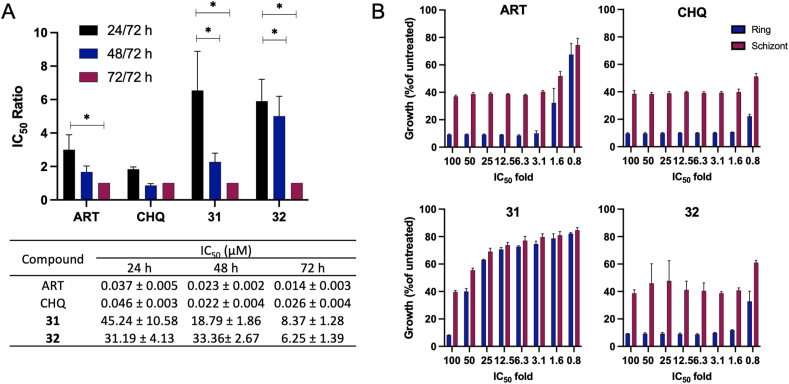
(A) The IC_50_ speed assay results for artemisinin (ART), chloroquine (CHQ), and Compounds **31** and **32**. The significance of the differences in IC_50_ ratio between tested compounds was analyzed using two-way ANOVA with Tukey's multiple comparisons test. (B) the stage-specific impacts of ART, CHQ, and Compounds **31** and **32** on synchronized cultures of *P. falciparum* 3D7. The cultures were treated with eight different compound concentrations for 24 h. The effects of the compounds are quantified as the percentage growth at the specified developmental stage in comparison to an untreated control group. Each bar illustrates the mean ± standard deviation (SD) derived from three independent experiments.

The data presented in Fig. 4A, using the IC_50_ speed assay, demonstrates that ART and CHQ, along with Compounds **31** and **32**, display varied kinetics in inhibiting *P. falciparum* 3D7 growth over time. The IC_50_ values obtained at 24, 48, and 72 h for ART and CHQ show minimal deviation from the standard 72-h assay, indicating their fast-acting nature. Specifically, ART and CHQ exhibit a ratio close to 1 when comparing the 24-h IC_50_ to the 72-h value, underscoring their rapid inhibitory effects. In contrast, Compounds **31** and **32**, exhibited slow-acting properties, with compound **31** demonstrating a moderately faster action than compound **32**.

The stage specificity analysis presented in Fig. 4B further defines the actions of these compounds across ring and schizont stages. Both ART and CHQ demonstrate potency against ring and schizont stages, consistent with their known rapid-acting antimalarial profiles. The profiles of Compounds **31** and **32**, however, are more complex. Compound **31** exhibits a classic slow-acting profile, which only gains significant activity at elevated concentrations. Compound **32**'s profile is inconsistent; while the rate speed assay suggests a relatively slow action, its effect on the ring and schizont stages is comparable to those positive controls. Such a contradiction highlights the intricate nature of antimalarial interactions, suggesting that a compound like **32** might hinder parasite metabolism without causing immediate death. It is important to consider the limitations of the SYBR Green I assay in this context, especially its inability to distinguish between metabolically active or dead parasites (Wein et al., 2010).

**Fig. 5:**
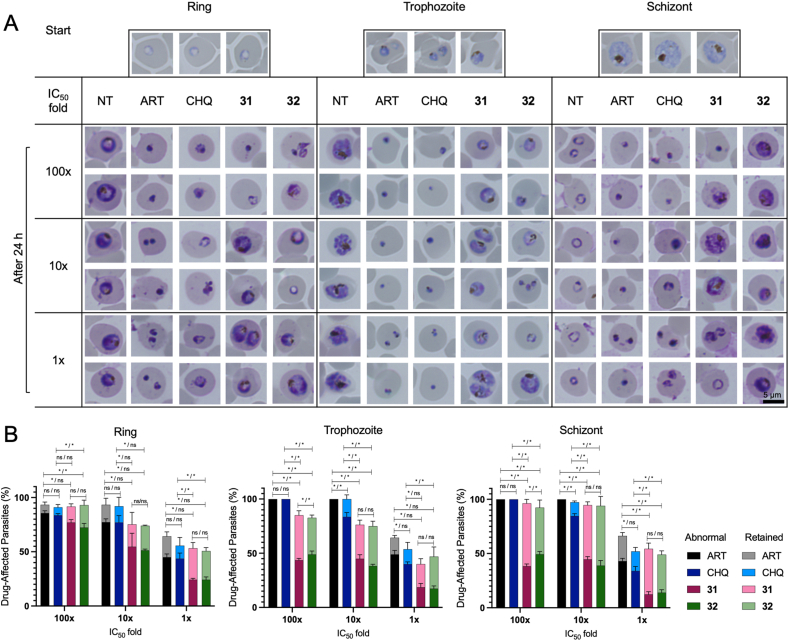
(A) Representative morphologies of parasites after a 24-h incubation period under different conditions, including no treatment (NT), artemisinin (ART), chloroquine (CHQ), and Compounds **31** and **32**. Each of these compounds was added at concentrations of 100-, 10-, and 1-fold their respective IC_50_ values. The experiments involved parasites at various stages of development, including the ring, trophozoite, and schizont stages. The scale bar provided is 5 μm. (B) The percentages of drug-affected parasites, encompassing both abnormal and retained morphologies, after exposure to ART, CHQ, and Compounds **31** and **32** for 24 h. The concentrations used were 100-, 10-, and 1-fold their respective IC_50_ values. The percentages are averages from triplicate wells, with error bars representing the standard deviations. Significance levels denoted by two symbols for abnormal/retained parasites indicate statistical significance (*, significant, *P* < 0.05; ns, not significant, *P* > 0.05). The significance of the differences in parasitemia levels between tested compounds was analyzed using one-way ANOVA with Tukey's multiple comparisons test.

Microscopic examinations of the thin blood smears prepared from *P. falciparum* 3D7 incubated with Compounds **31** and **32** were used to investigate the impacts of the compounds on the morphological changes of the parasite and parasitemia levels over time. The focus of the initial observation was to assess the specific effects of the tested compounds during the ring, trophozoite, and schizont stages. Notably, when the compounds were administered at concentrations 100 times their respective IC_50_ values, the parasites exhibited distinct arrest in progressing to subsequent cycle stages in contrast to the no treatment group (Fig. 5). Treatment with artemisinin and chloroquine at all concentrations led to the death and shrinkage of the parasite at all stages. Conversely, Compounds **31** and **32** prompted the emergence of an abnormal phenotype of ring-stage parasites (Fig. 5B Ring) alongside retained lifecycle phenotypes for the trophozoite and schizont stages (Fig. 5B Trophozoite and Schizont). Furthermore, treatment with Compounds **31** and **32** at 10- and 1-fold IC_50_ induced only partial inhibition of the life cycle, and significant differences in drug-affected parasites compared to the positive controls were observed (Fig. 5B).

The investigation into the effects of Compounds **31** and **32** on *P. falciparum* 3D7 was further focused on assessing changes in parasite morphology and parasitemia levels over a period of up to 144 h, with specific interest in the outcomes post-exposure and after removal of the compounds. Initial observations during the first 72 h of incubation revealed that Compounds **31** and **32** did not significantly alter the life cycle or phenotype of the parasite compared to untreated controls, maintaining a similar morphological appearance. Despite a marked increase in parasitemia levels at 48 and 72 h, indicative of ongoing parasite replication, the presence of Compounds **31** and **32** was associated with significantly lower parasitemia levels compared to untreated controls. This finding suggests a partial inhibitory effect on parasite growth, classifying both compounds as having potential static effects during this early phase of exposure. Extended observation revealed distinct outcomes for each compound. After 72 h of exposure to Compound **31**, not only did the inhibitory effect on parasite growth persist, but there was also a noticeable morphological alteration, with parasites appearing as shrunken, dot-like entities within erythrocytes. This morphological change, coupled with reduced parasitemia levels, hints at a possible cidal effect of Compound **31**, as it impaired the parasite's ability to maintain its normal structure and proliferate. In contrast, Compound **32** exhibited a more nuanced effect. While it effectively suppressed parasite growth to levels below that of the untreated control, it allowed for the maintenance of normal parasite morphology at later stages (120 and 144 h) in both unwashed and washed groups. This observation suggests that Compound **32** possesses a predominantly static effect, capable of inhibiting parasite proliferation without necessarily killing the parasites or significantly disrupting their developmental stages. The persistence of inhibitory activity against parasite growth by Compound **32**, especially in the unwashed group, and its ability to maintain parasitemia levels in the washed group at its IC_80_ concentration, reinforces its role as an inhibitor. However, the lack of morphological disruption suggests that while Compound **32** can control parasite growth, its mode of action may not lead to outright parasite death, differentiating its cidal versus static effects in comparison to Compound **31**.

### 3.4 Effects of compounds 31 and 32 on *P. Yoelii* 17XNL-infected mice

In the determination of the dose for in vivo assays in this study, a 20 mg/kg dosage was chosen, based on the requirements demonstrated by antimalarial drugs such as ART and CHQ. These drugs have been shown to require a range of 10–50 mg/kg in mouse models to cure infections (Simwela et al., 2020). The median point within this range was selected to maintain a balance between efficacy and safety. Furthermore, a modified Peter's suppression test was utilized to assess the efficacy of antimalarial drugs (Ager, 1984; Peters et al., 1975). The treatment and observation period were initially set for 4 days. However, due to the slow action of Compounds **31** and **32**, and the variable rates of parasite clearance, a modification was incorporated into the method: if no significant decrease in parasitemia levels was observed after this initial 4-day period, the treatment duration was extended, allowing for continued daily treatment up to a maximum of 10 days, avoiding bothersome effects. In the observation of chloroquine efficacy, a treatment duration of 7 days was required to decrease the parasitemia levels from the peak of infection (see supplementary data Figure S3A). A daily dose of 20 mg/kg of Compounds **31** or **32** over a 10-day period resulted in a significant reduction in parasitemia when compared to the untreated control group (Fig. 8). Importantly, there were no instances of mortality in the untreated control group and the groups treated with Compounds **31** and **32**, nor were reductions in body weight or hematocrit levels observed (Fig. 8). Furthermore, there were significantly fewer parasitemia peaks in both the Compound **31**-treated group (trial 1: 12.7%, trial 2: 15.8%) and the Compound **32**-treated group (trial 1: 12.7%, trial 2: 14.0%) than in the untreated group (trial 1: 21.7%, trial 2: 28.3%). These findings highlight the effect of the 20 mg/kg/day dosage of Compounds **31** and **32** in reducing parasitemia without causing side effects.

## 4 Discussion

The in vitro antiplasmodial activities of 48 derivatives of 2,5-diphenyloxazole were screened against *P. falciparum* 3D7. Among the tested compounds, 14 compounds, including the synthesized natural products Compounds **1**, **4**, **7,** and **10**, exhibited potent parasite multiplication inhibition (Table 1). Their synthesis accessibility prediction scores are listed in Table 2. Among the synthesized natural products, the IC_50_ values of the compounds were in the order of **7** < **4** < **1** < **10** in the case of 3D7 and **7** < **4** < **10** < **1** in the case of K1. These results indicate that both the number and position of hydroxy groups are important for antiplasmodial activity and suggest that hydroxy-substituted 2,5-diphenyloxazoles can effectively inhibit the growth of *P. falciparum* in vitro. The Compound **1** analogs with a modified oxazole core, **40** (thiazole), **42** (1,3,4-oxadiazole), **44** (1,3,4-thiadiazole), and **47** (positional isomer), exhibited slightly higher activity than **1**, indicating that modification of the oxazole core does alter antiplasmodial activity. Unfortunately, the increase in the inhibitory activity of these compounds was followed by an increase in the toxicity to HFFs. Furthermore, methylation of the phenolic hydroxy groups, as in Compounds **2**, **5**, **8**, **11**, **13**–**30**, **41**, **45**, and **46**, dramatically reduced the inhibitory activity. Acetate derivatives **3**, **6**, **9**, and **12** exhibited slightly better inhibitory profiles than their corresponding hydroxy derivatives. Interestingly, when only two of the hydroxy groups in Compound **1** were converted to methoxy groups (those at positions 2′ and 5′, Compound **31**), the inhibitory activity was retained, and the cytotoxicity was reduced. Moreover, conversion of the 2″-hydroxy in Compound **31** to a 2″-benzyloxy group (OBn, Compound **32**) gives similar inhibitory activity, while conversion at this position to lower alkyl groups (OMOM, Compound **33**; OEt, Compound **34**; or O-*n*Pr, Compound **35**) or to nitrogen-containing groups (NO_2_, Compound **36**; NH_2_, Compound **37**; N(CH_3_)_2_, Compound **38**; or NHAc, Compound **39**) reduces the inhibitory activity. The statistical SAR analysis underscores the impact of molecular descriptors on inhibitory activity. Key findings from this analysis highlight the increased capacity for hydrogen bonding, which enhances antimalarial efficacy. Additionally, the analysis revealed that compounds with higher TPSA are more effective. Conversely, a positive correlation between LogP and IC_50_ was observed, indicating that increased lipophilicity may be associated with decreased biological activity.

Among the 14 hit compounds, Compounds **31** and **32** emerged as the most potent, as evidenced by their superior selectivity index (SI) values, which serve as indicators of safety within in vitro systems. This conclusion is also supported by the drug-likeness profiles of both compounds. Therefore, we selected these two compounds for further in vitro and in vivo investigation. The IC_50_ speed and stage-specific assay reveal that ART and CHQ rapidly inhibit the growth of *P. falciparum* 3D7, with IC_50_ values consistent across 24, 48, and 72 h, as shown by ratios near unity. In contrast, Compounds **31** and **32** are slower in action, with **31** being moderately more efficient than **32**. Complementing this, stage specificity analysis shows that while ART and CHQ are potent across parasite stages, Compound **31** acts slower and requires higher concentrations to be effective. Compound **32**, albeit slower according to the rate speed assay, impacts both ring and schizont stages comparably to artemisinin and chloroquine. This finding suggests that Compound **32** may impede parasite metabolism in a way that does not align with the speed of action observed, an insight that points to the complexity of antimalarial efficacy beyond simple speed, involving a nuanced interplay with the parasite's life cycle stages. In the microscopy observation, after 24 h of exposure to lower concentrations of Compounds **31** and **32**, treatment resulted in a higher percentage of life cycle retention in the parasites rather than the induction of abnormal morphology, especially for the trophozoite and schizont stages (Fig. 5). This finding suggests that the compounds may not have a cidal effect on the parasites within this timeframe, particularly when administered at lower concentrations. Thus, we intended to examine the culture conditions during a longer incubation time frame.

**Fig. 6:**
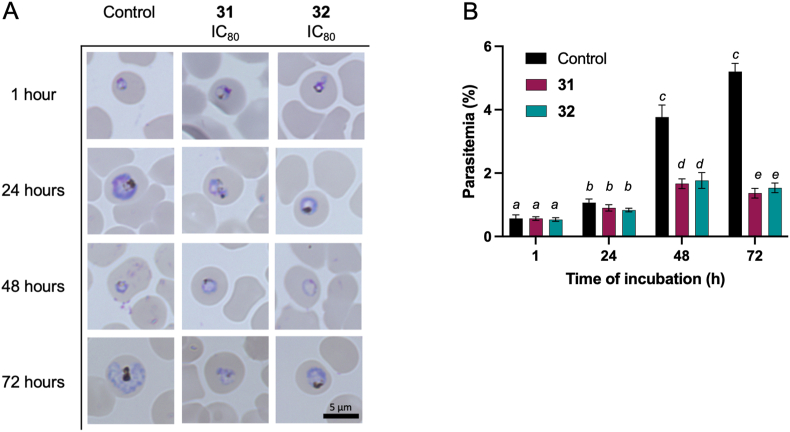
(A) Representative morphologies of parasites after various incubation periods, including 1, 24, 48, and 72 h, under different conditions: no treatment (untreated control), Compound **31** (IC_80_: 18.08 μM), and Compound **32** (IC_80_: 11.74 μM). The scale bar provided is 5 μm. (B) The parasitemia levels after incubation for 1, 24, 48, and 72 h under the same conditions mentioned above. The parasitemia levels given are the average values obtained from triplicate wells, and the error bars represent the standard deviations. Significance levels denoted by different letters indicate statistical significance (*P* < 0.05). The significance of differences in parasitemia levels between the Compound **31**- and Compound **32**-treated cultures compared to the untreated control for each group and at 1 h were analyzed using two-way ANOVA with Fisher's multiple comparisons test.

**Fig. 7:**
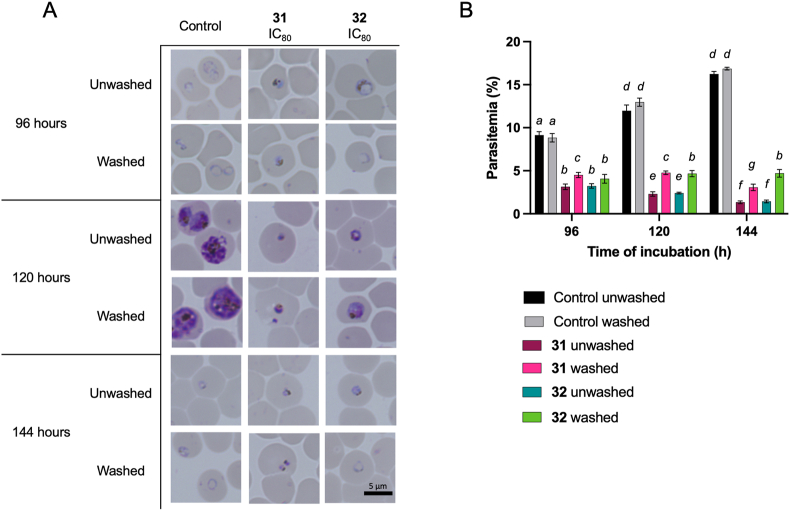
(A) Representative morphologies of parasites after 72 h of growth under different conditions: no treatment (untreated control), Compound **31** (IC_80_: 18.08 μM), and Compound **32** (IC_80_: 11.74 μM). The parasites were observed at subsequent time points, including 96, 120, and 144 h of incubation, in both the presence (unwashed) and absence (washed) of Compounds **31** and **32**. The scale bar provided is 5 μm. (B) The parasitemia levels after 72 h of growth and at subsequent time points (96, 120, and 144 h). The parasitemia levels are presented as the average values obtained from triplicate wells with error bars representing the standard deviations. Significance levels denoted by different letters indicate statistical significance (*P* < 0.05). The significance of the differences in parasitemia levels between the cultures treated with Compounds **31** and **32** compared to the untreated control for each group and at 96 h were analyzed using two-way ANOVA and Fisher's multiple comparisons test.

The differential impacts of Compounds **31** and **32** at their IC_80_ concentrations on *P. falciparum* 3D7 cultures reveal insights into their antimalarial mechanisms and potential therapeutic implications. Initially, both compounds allowed for the progression from the ring to the trophozoite stage within the first 24 h, suggesting a delayed onset of action that does not immediately halt parasite development (Fig. 6A). This initial similarity, however, diverged with further incubation, highlighting the different properties of each compound. Compound **31**'s ability to arrest parasite development at the trophozoite stage, coupled with the observation of shrunken parasite morphology after 72 h, underscores a profound impact not just on the parasite's growth but also on its developmental cycle. This effect signifies a disruption in the normal lifecycle progression of the parasite, which could be indicative of induced stress or damage mechanisms triggered by the compound. The lack of increase in parasitemia levels further suggests that Compound **31** might prevent erythrocyte reinvasion (Fig. 6B). In contrast, Compound **32**'s action appeared to be more conservative, with no marked alterations in parasite morphology or lifecycle progression, yet it effectively maintained lower parasitemia levels compared to controls. This observation suggests a mechanism that inhibits parasite replication or growth without directly inducing morphological changes or complete developmental arrest (Fig. 6B). The extended incubation experiments, distinguishing between continuous exposure and post-exposure effects (unwashed vs. washed groups), further define the compounds' impacts. Compound **31**'s sustained inhibition of parasite regrowth post-exposure indicates a long-lasting effect, potentially through mechanisms that irreversibly damage the parasite or significantly impede its ability to recover and proliferate after drug removal (Fig. 7A). This enduring impact, supported by the persistence of shrunken morphologies, points towards a potent cidal. On the other hand, the consistent inhibitory effect of Compound **32**, even after washing, albeit without a significant decrease in parasitemia levels in the washed group, indicates a different action. While not as dramatically effective in reducing parasite numbers post-exposure as Compound **31**, Compound **32**'s ability to suppress parasitemia levels significantly lower than untreated controls demonstrate a static effect (Fig. 7B). These observations collectively highlight the nuanced but distinct antimalarial properties of Compounds **31** and **32**. While both exhibit inhibitory effects at their IC_80_ concentrations within 72 h, their modes of action—cidal versus static—offer different therapeutic potentials.

**Fig. 8:**
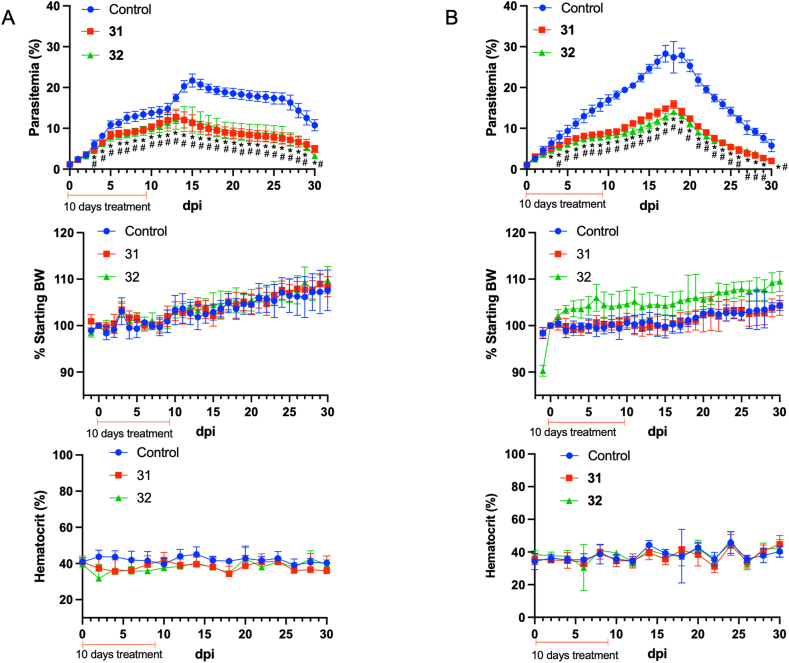
Effects of 10 days of treatment with Compounds **31** and **32** in C57BL/6 mice infected with *P. yoelii* 17XNL. Parasitemia levels, %starting body weight (BW) change, and hematocrit levels are presented for (A) trial 1 and (B) trial 2 following inoculation of 1 × 10^7^ infected erythrocytes. Each group consisted of six mice. Significance levels denoted by * and # indicate significant differences in parasitemia levels in the Compound **31**- and Compound **32**-treated mice compared to the untreated control mice, respectively. Statistical analysis was performed using two-way ANOVA followed by Tukey's multiple comparisons test with a threshold of *P* < 0.05.

To validate the in vitro inhibitory activity and assess the drug-likeness of Compounds **31** and **32**, an in vivo mouse model of malaria was employed using *P. yoelii* 17XNL. In this study, mice were administered Compound **31** or **32** intraperitoneally (i.p.) at a dosage of 20 mg/kg/day for 10 days. Treatment resulted in partial inhibition of parasitemia levels and significantly lower parasitemia peaks in both the **31**-treated group (trial 1: 12.7%, trial 2: 15.8%) and the **32**-treated group (trial 1: 12.7%, trial 2: 14.0%) compared to the untreated group (trial 1: 21.7%, trial 2: 28.3%). However, although both Compounds **31** and **32** were predicted to have good bioavailability based on SwissADME and pkCSM, the chosen dosage and route of administration proved to be insufficient to completely eliminate the parasite. This limitation can be attributed to various factors that can influence the bioavailability of administered compounds in vivo, such as bioaccessibility, matrix effects, transporter interactions, molecular transformations, and metabolizing enzymes (Rein et al., 2013). Consequently, it is imperative to perform a more comprehensive study to enhance compound bioavailability and achieve complete parasite eradication taking into account the complexities of in vivo pharmacokinetics and bioavailability.

In view of the diverse biological activities exhibited by oxazole compounds, which include anticancer (Kulkarni et al., 2021), anti-inflammatory (Meenakshi et al., 2023), antimicrobial (Speri et al., 2020), and neuroprotective effects (Luo et al., 2023), it is reasonable to anticipate that these compounds target a variety of proteins. An example within the 2,5-diphenyloxazole family is 2-(3′-hydroxyphenyl)-5-(4″-hydroxyphenyl)-oxazole, which has been identified as a promising inhibitor of 17β-hydroxysteroid dehydrogenase type 1 (17-β-HSD1) in humans (Bey et al., 2008). This enzyme holds significance as a novel target in mammary tumors and endometriosis due to its role in the catalytic conversion of estrone (E1) into estradiol (E2). Furthermore, in terms of antiplasmodial, the structural similarities between the compounds in this study suggest their potential involvement in the terpenoid-steroid biosynthesis pathway or metabolism within *Plasmodium* spp. For instance, the methylerythritol phosphate (MEP) pathway is essential for cellular isoprenoid biosynthesis in eukaryotes, including *P. falciparum* (Guggisberg et al., 2014). To the best of our knowledge, there have been no previous reports on the potential antiplasmodial drug targets of 2,5-diphenyloxazoles.

In summary, the 2,5-diphenyloxazole derivatives here displayed potent antiplasmodial activity, with some derivatives demonstrating selective inhibition in vitro. However, the in vivo study yielded only partial inhibition of parasite growth. While these activity levels may not compare favorably with current frontrunners in antimalarial research exhibiting sub-nanomolar activities, it's important to consider the early stage of this discovery process. The identification of these compounds serves as a preliminary step, with the understanding that further optimization and structural modification are necessary to enhance their potency and safety profiles. Additionally, these compounds may possess other advantageous pharmacological properties not fully explored in this study, such as a unique mechanism of action or favorable pharmacokinetic properties, which could warrant further investigation despite the current potency limitations. To gain deeper insights into the efficacy and inhibitory effects of these derivatives, further investigations employing alternative administration methods, varying doses, or structural modification to enhance the pharmacokinetic properties are warranted. Moreover, it is important to note that the current research primarily focused on blood-stage *Plasmodium* spp. Exploring other life stages, vector transmission, or potential drug targets may prove beneficial to broaden the utility of 2,5-diphenyloxazoles as antiplasmodial agents. Expanding the scope of research to encompass these aspects could uncover additional avenues for effectively combating malaria.

## CRediT authorship contribution statement

**Nanang R. Ariefta:** Data curation, Formal analysis, Investigation, Methodology, Visualization, Writing – original draft. **Koichi Narita:** Investigation, Methodology, Resources. **Toshihiro Murata:** Investigation, Methodology, Resources. **Yoshifumi Nishikawa:** Conceptualization, Funding acquisition, Project administration, Supervision, Validation, Writing – review & editing.

## Declaration of competing interest

The authors declare no conflicts of interest.
