# Supplementary material for: Evaluation of the antiplasmodial efficacy of synthetic 2,5-diphenyloxazole analogs of compounds naturally derived from Oxytropis lanata
Source: Int J Parasitol Drugs Drug Resist. 2024 Apr 18;25:100540. doi: 10.1016/j.ijpddr.2024.100540 (PMC11067372; doi:10.1016/j.ijpddr.2024.100540)
Supplement: Multimedia component 1 [file mmc1.docx]

Supplementary Data for

**Evaluation of Antiplasmodial Efficacy of Synthetic 2,5-Diphenyloxazoles Analogous to Naturally-Derived Compounds from *Oxytropis lanata***

Nanang R. Ariefta^1^, Koichi Narita^2^, Toshihiro Murata^2^, Yoshifumi Nishikawa^1,^ *

^1^ National Research Center for Protozoan Diseases, Obihiro University of Agriculture and Veterinary Medicine, Inada-cho, Obihiro 080-8555, Japan

^2^ Faculty of Pharmaceutical Sciences, Tohoku Medical and Pharmaceutical University, 4-4-1 Komatsushima, Aoba-ku, Sendai 981-8558, Japan

*Correspondence to:

Prof. Yoshifumi Nishikawa

National Research Center for Protozoan Diseases,

Obihiro University of Agriculture and Veterinary Medicine,

Inada-cho, Obihiro 080-8555, Japan

E-mail: [nisikawa@obihiro.ac.jp](mailto:nisikawa@obihiro.ac.jp)

**Table S1**. Summary of the IC_50_ of 2,5-diphenyloxazoles against *P. falciparum* 3D7.

| Compound Number | Molecular weight | IC_50_ (μM) | | Compound Number | Molecular weight | IC_50_ (μM) | |
| --- | --- | --- | --- | --- | --- | --- | --- |
|  |  | Mean | SD |  |  | Mean | SD |
| **1** | 269.26 | 11.03 | 2.69 | **25** | 281.31 | 29.94 | 3.12 |
| **2** | 311.34 | >200.00 | - | **26** | 281.31 | >200.00 | - |
| **3** | 395.37 | 9.22 | 1.68 | **27** | 311.34 | 106.33 | 25.14 |
| **4** | 269.26 | 6.20 | 4.23 | **28** | 341.36 | 124.65 | 60.07 |
| **5** | 311.34 | >200.00 | 0.00 | **29** | 341.36 | 24.92 | 7.86 |
| **6** | 395.37 | 13.38 | 9.43 | **30** | 295.34 | >200.00 | - |
| **7** | 253.26 | 3.85 | 1.87 | **31** | 297.31 | 8.37 | 1.28 |
| **8** | 281.31 | >200.00 | - | **32** | 387.44 | 6.25 | 1.39 |
| **9** | 337.33 | 3.38 | 1.46 | **33** | 341.36 | 18.20 | 1.69 |
| **10** | 285.26 | 12.65 | 7.63 | **34** | 325.36 | >200.00 | - |
| **11** | 341.36 | 15.03 | 2.51 | **35** | 339.39 | 27.67 | 3.58 |
| **12** | 453.4 | 10.23 | 4.09 | **36** | 326.31 | 39.60 | 9.43 |
| **13** | 281.31 | >200.00 | - | **37** | 296.33 | 36.64 | 5.30 |
| **14** | 331.37 | 62.37 | 1.50 | **38** | 324.38 | 54.22 | 22.95 |
| **15** | 311.34 | 74.81 | 3.21 | **39** | 338.36 | >200.00 | - |
| **16** | 311.34 | 37.92 | 2.15 | **40** | 285.32 | 3.95 | 1.38 |
| **17** | 341.36 | >200.00 | - | **41** | 327.4 | 15.46 | 0.41 |
| **18** | 371.39 | 51.05 | 29.42 | **42** | 270.24 | 9.53 | 3.15 |
| **19** | 281.31 | 46.27 | 6.47 | **43** | 312.33 | 16.92 | 4.45 |
| **20** | 281.31 | >200.00 | - | **44** | 286.31 | 6.41 | 1.16 |
| **21** | 311.34 | 19.33 | 0.39 | **45** | 328.39 | >200.00 | - |
| **22** | 311.34 | 26.66 | 5.20 | **46** | 311.34 | 44.69 | 15.82 |
| **23** | 341.36 | 12.71 | 1.77 | **47** | 269.26 | 4.63 | 1.76 |
| **24** | 371.39 | 67.98 | 7.29 | **48** | 401.42 | 8.01 | 3.20 |

The reported values are presented as the average derived from three independent experiments. The hit compounds are highlighted.

**Table S2**. The SwissADME calculated topological descriptors of 2,5-diphenyloxazoles used in this study.

| **ID** | **smiles** | **IC_50_** | **logIC_50_** | **MW** | **NumHAcceptors** | **NumHDonors** | **NumRotatableBonds** | **TPSA** | **LogP** |
| --- | --- | --- | --- | --- | --- | --- | --- | --- | --- |
| 1 | Oc3cccc(c2ncc(c1ccccc1O)o2)c3O | 11.02528 | 1.04239 | 269.25 | 5 | 3 | 2 | 86.72 | 2.07 |
| 2 | COc1ccccc1c3cnc(c2cccc(OC)c2OC)o3 | >200 | 2.30103 | 311.33 | 5 | 0 | 5 | 53.72 | 3.25 |
| 3 | CC(=O)Oc1ccccc1c3cnc(c2cccc(OC(C)=O)c2OC(C)=O)o3 | 9.223428 | 0.964892 | 395.36 | 8 | 0 | 8 | 104.93 | 2.97 |
| 4 | Oc3ccc(O)c(c2ncc(c1ccccc1O)o2)c3 | 6.199832 | 0.79238 | 269.25 | 5 | 3 | 2 | 86.72 | 2.31 |
| 5 | COc3ccc(OC)c(c2ncc(c1ccccc1OC)o2)c3 | >200 | 2.30103 | 311.33 | 5 | 0 | 5 | 53.72 | 3.32 |
| 6 | CC(=O)Oc3ccc(OC(C)=O)c(c2ncc(c1ccccc1OC(C)=O)o2)c3 | 13.38409 | 1.126589 | 395.36 | 8 | 0 | 8 | 104.93 | 3.4 |
| 7 | Oc3ccc(O)c(c2cnc(c1ccccc1)o2)c3 | 3.847693 | 0.5852 | 253.25 | 4 | 2 | 2 | 66.49 | 2.35 |
| 8 | COc3ccc(OC)c(c2cnc(c1ccccc1)o2)c3 | >200 | 2.30103 | 281.31 | 4 | 0 | 4 | 44.49 | 3.36 |
| 9 | CC(=O)Oc3ccc(OC(C)=O)c(c2cnc(c1ccccc1)o2)c3 | 3.37612 | 0.528418 | 337.33 | 6 | 0 | 6 | 78.63 | 3.32 |
| 10 | Oc3ccc(O)c(c2cnc(c1cc(O)ccc1O)o2)c3 | 12.64928 | 1.102066 | 285.25 | 6 | 4 | 2 | 106.95 | 1.97 |
| 11 | COc3ccc(OC)c(c2cnc(c1cc(OC)ccc1OC)o2)c3 | 15.03333 | 1.177055 | 341.36 | 6 | 0 | 6 | 62.95 | 3.67 |
| 12 | CC(=O)Oc3ccc(OC(C)=O)c(c2cnc(c1cc(OC(C)=O)ccc1OC(C)=O)o2)c3 | 10.22864 | 1.009818 | 453.4 | 10 | 0 | 10 | 131.23 | 3.25 |
| 13 | COc1ccccc1c3cnc(c2ccccc2OC)o3 | >200 | 2.30103 | 281.31 | 4 | 0 | 4 | 44.49 | 3.2 |
| 14 | COc4cccc(c3ncc(c2ccc1ccccc1c2)o3)c4OC | 62.36667 | 1.794953 | 331.36 | 4 | 0 | 4 | 44.49 | 3.64 |
| 15 | COc3cccc(c2cnc(c1cccc(OC)c1OC)o2)c3 | 74.80667 | 1.87394 | 311.33 | 5 | 0 | 5 | 53.72 | 3.53 |
| 16 | COc3ccc(c2cnc(c1cccc(OC)c1OC)o2)cc3 | 37.92 | 1.578868 | 311.33 | 5 | 0 | 5 | 53.72 | 3.52 |
| 17 | COc3ccc(OC)c(c2cnc(c1cccc(OC)c1OC)o2)c3 | >200 | 2.30103 | 341.36 | 6 | 0 | 6 | 62.95 | 3.56 |
| 18 | COc3cccc(c2ncc(c1ccc(OC)c(OC)c1OC)o2)c3OC | 51.05333 | 1.708024 | 371.38 | 7 | 0 | 7 | 72.18 | 3.89 |
| 19 | COc3cccc(c2cnc(c1cccc(OC)c1)o2)c3 | 46.27 | 1.665299 | 281.31 | 4 | 0 | 4 | 44.49 | 3.41 |
| 20 | COc3ccc(c2cnc(c1ccc(OC)cc1)o2)cc3 | >200 | 2.30103 | 281.31 | 4 | 0 | 4 | 44.49 | 3.38 |
| 21 | COc3ccc(c2ncc(c1ccccc1OC)o2)c(OC)c3 | 19.32667 | 1.286157 | 311.33 | 5 | 0 | 5 | 53.72 | 3.43 |
| 22 | COc1ccccc1c3cnc(c2c(OC)cccc2OC)o3 | 26.66 | 1.42586 | 311.33 | 5 | 0 | 5 | 53.72 | 3.13 |
| 23 | COc3cccc(c2cnc(c1cccc(OC)c1OC)o2)c3OC | 12.70667 | 1.104032 | 341.36 | 6 | 0 | 6 | 62.95 | 3.73 |
| 24 | COc3cccc(c2cnc(c1ccc(OC)c(OC)c1OC)o2)c3OC | 67.98 | 1.832381 | 371.38 | 7 | 0 | 7 | 72.18 | 3.72 |
| 25 | COc3cccc(c2ncc(c1ccccc1)o2)c3OC | 29.94 | 1.476252 | 281.31 | 4 | 0 | 4 | 44.49 | 3.19 |
| 26 | COc3cccc(c2ncc(c1ccccc1OC)o2)c3 | >200 | 2.30103 | 281.31 | 4 | 0 | 4 | 44.49 | 3.22 |
| 27 | COc3ccc(OC)c(c2cnc(c1ccccc1OC)o2)c3 | 106.33 | 2.026656 | 311.33 | 5 | 0 | 5 | 53.72 | 3.52 |
| 28 | COc3cccc(c2ncc(c1c(OC)cccc1OC)o2)c3OC | 124.65 | 2.095692 | 341.36 | 6 | 0 | 6 | 62.95 | 3.57 |
| 29 | COc3ccc(c2cnc(c1cccc(OC)c1OC)o2)c(OC)c3 | 24.92333 | 1.396606 | 341.36 | 6 | 0 | 6 | 62.95 | 3.56 |
| 30 | COc3cccc(c2ncc(c1ccccc1C)o2)c3OC | >200 | 2.30103 | 295.33 | 4 | 0 | 4 | 44.49 | 3.4 |
| 31 | COc3cccc(c2ncc(c1ccccc1O)o2)c3OC | 8.367249 | 0.922583 | 297.31 | 5 | 1 | 4 | 64.72 | 2.97 |
| 32 | COc4cccc(c3ncc(c1ccccc1OCc2ccccc2)o3)c4OC | 6.253872 | 0.796149 | 387.43 | 5 | 0 | 7 | 53.72 | 3.79 |
| 33 | COCOc1ccccc1c3cnc(c2cccc(OC)c2OC)o3 | 18.20333 | 1.260151 | 341.36 | 6 | 0 | 7 | 62.95 | 3.46 |
| 34 | CCOc1ccccc1c3cnc(c2cccc(OC)c2OC)o3 | >200 | 2.30103 | 325.36 | 5 | 0 | 6 | 53.72 | 3.52 |
| 35 | CCCOc1ccccc1c3cnc(c2cccc(OC)c2OC)o3 | 27.66667 | 1.441957 | 339.39 | 5 | 0 | 7 | 53.72 | 3.7 |
| 36 | COc3cccc(c2ncc(c1ccccc1N(=O)=O)o2)c3OC | 39.60333 | 1.597732 | 326.3 | 6 | 0 | 5 | 90.31 | 2.51 |
| 37 | COc3cccc(c2ncc(c1ccccc1N)o2)c3OC | 36.64 | 1.563955 | 296.32 | 4 | 1 | 4 | 70.51 | 2.9 |
| 38 | COc3cccc(c2ncc(c1ccccc1N(C)C)o2)c3OC | 54.21667 | 1.734133 | 324.37 | 4 | 0 | 5 | 47.73 | 3.4 |
| 39 | COc3cccc(c2ncc(c1ccccc1NC(C)=O)o2)c3OC | >200 | 2.30103 | 338.36 | 5 | 1 | 6 | 73.59 | 3.28 |
| 40 | Oc3cccc(c2ncc(c1ccccc1O)s2)c3O | 3.953222 | 0.596951 | 285.32 | 4 | 3 | 2 | 101.82 | 2.53 |
| 41 | COc1ccccc1c3cnc(c2cccc(OC)c2OC)s3 | 15.46 | 1.189209 | 327.4 | 4 | 0 | 5 | 68.82 | 3.39 |
| 42 | Oc3cccc(c2nnc(c1ccccc1O)o2)c3O | 9.533501 | 0.979252 | 270.24 | 6 | 3 | 2 | 99.61 | 1.65 |
| 43 | COc1ccccc1c3nnc(c2cccc(OC)c2OC)o3 | 16.92227 | 1.228459 | 312.32 | 6 | 0 | 5 | 66.61 | 3.03 |
| 44 | Oc3cccc(c2nnc(c1ccccc1O)s2)c3O | 6.405644 | 0.806563 | 286.31 | 5 | 3 | 2 | 114.71 | 2.2 |
| 45 | COc1ccccc1c3nnc(c2cccc(OC)c2OC)s3 | >200 | 2.30103 | 328.39 | 5 | 0 | 5 | 81.71 | 3.3 |
| 46 | COc1ccccc1c3coc(c2cccc(OC)c2OC)n3 | 44.69 | 1.65021 | 311.33 | 5 | 0 | 5 | 53.72 | 3.4 |
| 47 | Oc3cccc(c2nc(c1ccccc1O)co2)c3O | 4.632697 | 0.665834 | 269.25 | 5 | 3 | 2 | 86.72 | 2.18 |
| 48 | COc3ccc(c2cnc(c1ccc(OC)c(OC)c1OC)o2)c(OC)c3OC | 8.01405 | 0.903852 | 401.41 | 8 | 0 | 8 | 81.41 | 4.13 |

**Table S3.** Criteria for normal and abnormal parasite morphologies after 24 h exposure of the drugs.

| Start at | Ring | Trophozoite | Schizont |
| --- | --- | --- | --- |
| Normal Morphologies | Develop to trophozoite stage. The parasite grows inside the red blood cell and undergoes nuclear division. The trophozoite should have a single nucleus with a visible chromatin dot, and the cytoplasm should be evenly distributed throughout the cell. | Develop to schizont stage. The parasite undergoes multiple rounds of nuclear division and produces multiple daughter cells. The schizont should have multiple nuclei arranged in a rosette pattern, and the cytoplasm should be evenly distributed throughout the cell. | Develop to ring stage. The parasite in the small ring-shaped structure inside the red blood cell. The ring should be uniform in size, with a centrally located nucleus and a single chromatin dot. |
| Abnormal Morphologies | Irregular shape, multiple nuclei, uneven distribution of cytoplasm, or parasite shrinkage. | Irregular shape, incomplete nuclear division, uneven distribution of cytoplasm, or parasite shrinkage. | Multiple nuclei, enlarged size, irregular shape, or parasite shrinkage. |
| Stage retained | Retained at ring stage | Retained at trophozoite stage | Retained at schizont stage |


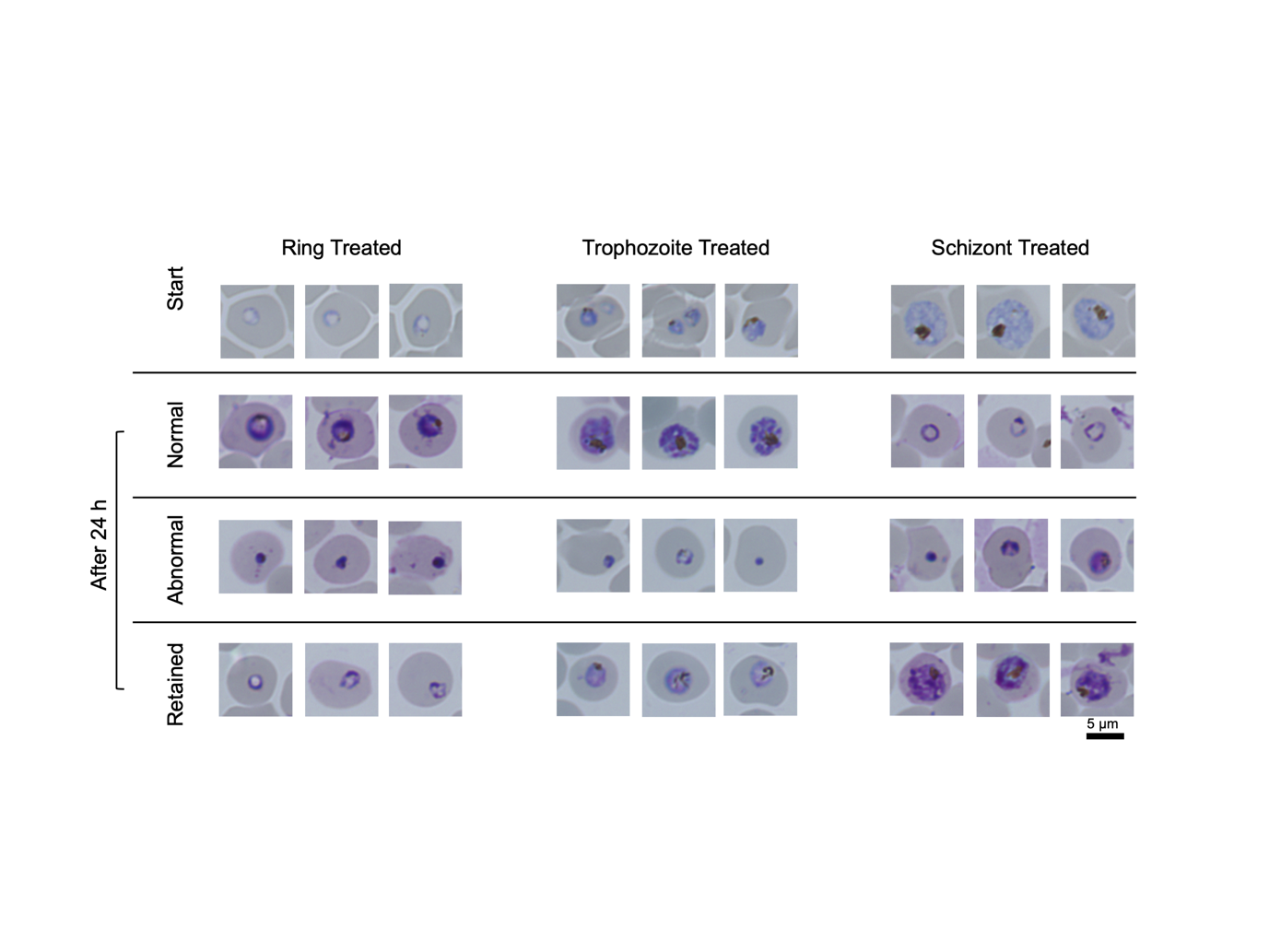


**Figure S1**. Representative pictures of normal and abnormal parasite morphologies based on the criteria on Table S3.

**Figure S2**. The hemolysis rate of artemisinin (ART), chloroquine (CHQ), **31**, and **32** at range of 1000-7.812 μM (two times dilution, 8 concentrations).

**Figure S3**. Parasitemia levels after treatment with (A) 7 times 20 mg/kg/day chloroquine , (B) Trial 1, and (C) Trial 2, 10 times 20 mg/kg/day **31** and **32**, following the inoculation of 1 × 10^7^ infected erythrocytes of *P. yoelii* 17XNL. Each group consisted of six mice. (* or #) The significance of differences in the level of parasitemia in the treated mice compared with the control mice. The significance of differences was analyzed by two-way ANOVA followed by Tukey’s multiple-comparison test (*P* < 0.05).

The phyton code used in the SAR analysis within Jupyter Notebook.
